# A 646C > G (rs41423247) polymorphism of the glucocorticoid receptor as a risk factor for hyperglycaemia diagnosed in pregnancy—data from an observational study

**DOI:** 10.1007/s00592-021-01799-3

**Published:** 2021-10-14

**Authors:** Agnieszka Zawiejska, Anna Bogacz, Rafał Iciek, Agnieszka Lewicka-Rabska, Maciej Brązert, Przemysław Mikołajczak, Jacek Brązert

**Affiliations:** 1 Chair of Medical Education, Department of Medical Simulation, Poznan University of Medical Sciences, Poznan, Poland; 2 Institute of Natural Fibers and Medicinal Plants, National Research Institute, Poznan, Poland; 3 Department of Obstetrics and Women’s Diseases, Poznan University of Medical Sciences, Poznan, Poland; 4 Department of Hypertension, Angiology and Internal Medicine, Poznan University of Medical Sciences, Poznan, Poland; 5 Department of Infertility and Reproductive Endocrinology, Poznan University of Medical Sciences, Poznan, Poland; 6 Chair of Pharmacology, Poznan University of Medical Sciences, Poznan, Poland

**Keywords:** Hyperglycaemia detected in pregnancy, Gestational diabetes mellitus, Diabetes diagnosed in pregnancy, Glucocorticoid receptor, rs41423247 polymorphism

## Abstract

**Aim:**

Hyperglycaemia diagnosed in pregnancy (HiP) is a serious and frequent complication of pregnancy, increasing the risk for adverse maternal and neonatal outcomes. Investigate whether allelic variations of the glucocorticoid receptor are related to an increased risk of HiP.

**Method:**

The following polymorphisms of the glucocorticoid receptor (*GR*) were investigated in the cohort study of *N* = 197 pregnant women with HiP and *N* = 133 normoglycemic pregnant controls: 646C > G (rs41423247), N363S (rs6195), ER23/22EK (rs6190, rs6189).

**Results:**

A *GG* variant of the rs41423247 polymorphism was associated with a significantly higher risk for HiP: OR 1.94 (1.18; 3.18), *p* = 0.009. The relationship remained significant after controlling for maternal age and prepregnancy BMI: OR 3.09 (1.25; 7.64), *p* = 0.014.

**Conclusions:**

The allelic *GG* variant of the 646C > G (rs41423247) polymorphism is associated with an increased risk for hyperglycaemia in pregnancy.

## Introduction

Glucocorticoids and their receptor (GR) provide an essential pleiotropic regulatory system for mammalian metabolism. In humans, an excess of the GR ligands—both exogenous and endogenous—is a well-known reason for metabolic derangement, particularly for obesity, insulin resistance and hyperglycaemia.

Available evidence describes an association between *GR* gene variants and altered response to glucocorticoids in the target tissues due to the altered transcriptional activity of the GR [1]. According to the studies by Russcher [2], carriers of the ER22/23EK and 3669A > G variant presented a reduced transcriptional activity of the GR, which might translate into a reduced risk of T2DM and cardiovascular disorders as a result of a relative insensitivity to endogenous glucocorticosteroids [1].

In turn, studies on the other variants of the receptor, Bcl I and N363S, linked these variants to increased sensitivity to endogenous glucocorticosteroids [1]. Additionally, studies performed in cohorts from the UK and the Netherlands translated these genetic findings into metabolically less favourable phenotypes of increased BMI or increased WHR in the adult carriers of the N363S polymorphism [3, 4].

Such observations suggest a relationship between the genetic variability of the *GR* and non-communicable disorders. However, translation of these findings into clinical practice has proven to be difficult. Studies on clinical phenotypes related to the polymorphisms of the *GR* in several national cohorts bring forth inconclusive findings*.* While associations between allelic variants of the *GR* and biomarkers of increased metabolic risk—mostly insulin resistance or hyperglycaemia—were noted, these results differ across age, nationality and sex [5]. Moreover, described associations regard small subsets of the participants due to a low prevalence of the variants in the studied cohorts [6]. These findings are important for understanding the patomechanisms of metabolic disorders but provide little explanation regarding public health.

Clinical data indicate that pregnant women with hyperglycaemia detected in pregnancy (HiP) share their risk factors profile with the population with T2DM [7]. Recent data confirm a shared pathomechanism involving a combination of pancreatic beta-cell dysfunction and target tissues’ insulin resistance [8]. We noted genetic and environmental factors tightly interwoven in both diseases, contributing to the heterogeneity of clinical presentation, response to treatment or a wide range of short-term or long-term consequences [9].

Hyperglycaemia diagnosed in pregnancy, also known as gestational diabetes (GDM) or diabetes diagnosed in pregnancy (DiP), is currently among the leading risks for pregnant women and their foetuses. The latest edition of the IDF Diabetes Atlas reports that a global total of 14.5% of all live births are born to women with GDM or DiP [10]. Advancing procreative age and lifestyle changes resulting in increasing maternal BMI are the best-studied factors contributing to the disease’s rising prevalence. HiP is commonly acknowledged as a risk factor for serious foetal and intrapartum complications leading to increased perinatal morbidity and mortality [11]. For mothers, HiP increases the risk for pregnancy-related metabolic disorders, such as gestational hypertension, excessive gestational weight gain, or postpartum body weight retention, all of which are now considered clinical manifestations of metabolic syndrome [12, 13].

The genetic aspect of T2DM is well studied. From the earliest reports identifying the first candidate genes for the disease studying affected families, novel high-output genome analysis techniques like GWAS or NGS identified hundreds of genetic variants associated with an increased risk of the disease. A recent study applied bioinformatics to perform functional analysis of 981 T2DM-associated, differently expressed genes [14]. The researchers used GO enrichment analysis, the Kyoto Encyclopaedia of Genes and Genomes pathway analysis and the protein–protein interaction network to identify a limited number of candidate genes considered to be of critical importance for initiation and progression of the disease. However, functional studies necessary to explain a causal relationship between genetic susceptibility and pathomechanisms of hyperglycaemia are still scarce [15, 16].

On the contrary, studies on the genetic background of HiP represent a relatively new area of research, with a limited, albeit rising amount of evidence available [17, 18]. The majority of available publications confirmed a positive association between the general risk for GDM and T2DM candidate genes that were widely studied in the general population: *TCF7L2*, *GCK*, *KCNJ11*, *CDKAL1* and *MTNR1B* [17, 19]. Our study in a single-site Polish population reported an association between a genetic variation in the *IRS-1* gene and HiP diagnosed in early pregnancy [20]. However, available studies primarily report data from small, single-site or national cohorts. Moreover, no available studies investigated the role of genetic variants of the GR gene in the development of hyperglycaemia in pregnancy.

We hypothesized that hyperglycaemia diagnosed in pregnancy is associated with a different distribution of the *GR* genotypes than that of the normoglycemic pregnant population. The aim of our study is to investigate whether any genetic variant of the *GR* receptor could be a risk factor for developing hyperglycaemia in pregnancy. To address our research question, we reviewed the literature and selected SNPs for which there is a considerable body of evidence from clinical studies linking these variants to various aspects of insulin resistance, obesity and metabolic syndrome. We also aimed at testing both SNPs associated with increased sensitivity to endogenous glucocorticoid and those associated with decreased sensitivity to the hormone.

## Materials and methods

To answer our research question, we designed an observational study.

All patients referred to the Department of Obstetrics and Women’s Diseases—an academic, tertiary-level care referral unit for high-risk pregnancies—for further treatment for hyperglycaemia diagnosed in pregnancy (HiP) were considered eligible for participation in the study. The inclusion criteria were as follows: singleton, viable pregnancy and no history of type 1 or type 2 diabetes. Women treated for insulin resistance before pregnancy, lacking data regarding their glucose values at the diagnosis of HiP or unwilling to participate were excluded from the study.

Finally, *N* = 197 women with HiP were enrolled on the study. Hyperglycaemia in pregnancy was diagnosed using the WHO-IADPSG criteria [21]. We also applied the glycaemic thresholds recommended by the guidelines to discriminate between gestational diabetes (GDM) and diabetes diagnosed in pregnancy (DiP) [21]. Data regarding OGTT and maternal prepregnancy anthropometrics were retrospectively retrieved from maternal documentation upon the first admission to the unit.

The controls (*N* = 133) were recruited among the pregnant women without a history of HiP or hypertensive disorders in pregnancy and who were admitted for delivery in singleton, live, full-term pregnancies. Data regarding maternal anthropometrics were retrieved from maternal documentation. The protocol was reviewed and approved by the Bioethical Committee at the University of Medical Sciences in Poznan (Protocol No: 646/09). All participants gave informed consent for participation in the study.

Genomic DNA isolation from venous blood samples was performed using a commercial kit QIAamp DNA Blood Mini Kit (Qiagen, Germany) following the manufacturer’s protocol. DNA purity and concentration were measured using an EPOCH spectrophotometer (Biokom). The analysis of the GR polymorphisms (rs41423247, rs6195, rs6189, rs6190) was determined by the real-time PCR method using LightCycler® 480 (Roche Diagnostics). Fluorescent dye-labelled hybridization probes were used for genotyping. A set of LightSNiPrs6189, LightSNiPrs6190, LightSNiPrs6195 and LightSNiPrsS41423247 (TibMolbiol, Germany) for GR polymorphisms contained appropriate concentrations of specific primers and probes for the amplified fragment and were prepared according to the manufacturer’s instructions. Each PCR cycle comprised a denaturation step at 95 °C for 10 s, an annealing step at 60 °C for 10 s and an elongation step at 72 °C for 15 s (45 cycles). The analysis of the results was based on the melting curve using LightCycler® 480 Basic Software.

Statistical analysis was performed using SPSS version 17.0 (IBM, Armonk, New York, USA). The genotypes’ expected frequency was calculated using the Hardy–Weinberg equation, which was compared with the values observed using the Pearson *χ*^2^ test. The *p* value of < 0.05 was considered statistically significant.

To investigate clinical factors associated with hyperglycaemia in pregnancy in our cohort, we used multivariate logistic regression analysis with bivariate logistic regression as a screening test. Analysis of ROC and logistic regression was performed with MedCalc version 19.8 (Ostend, Belgium). Maternal characteristics significantly related to the outcome, such as HiP status in the bivariate analysis (*p* < 0.10), were entered into the multivariate model using a forward step method. A *p* value of < 0.05 was considered to be statistically significant.

## Results

Characteristics of the cohort are summarised in Table 1. HiP was diagnosed in 59.7% of the cohort. A total of 26.8% of these patients had early HiP (eHiP), meaning their disease was diagnosed before the 24th week of gestation. Women with HiP were significantly older and heavier as compared to the controls [maternal age in years: 32.0 (28.0; 35.0) vs. 29.0 (26.0; 32.5) *p* < 0.001, prepregnancy body weight in kg: 84.0 (70.0; 99.0) vs. 61.0 (53.0; 68.0) *p* < 0.0001, prepregnancy BMI in kg/m^2^: 30.9 (25.8; 35.8) vs. 21.6 (19.5; 24.3) *p* < 0.001)].

**Table 1:** Characteristics of the cohort; data given as median (interquartile range)

Parameter	Median (interquartile range)
Maternal age (years)	31.0 (28.0; 35.0)
Maternal prepregnancy body weight (kg)	72.5 (60.7; 91.2)
Prepregnancy BMI (kg/m^2^)	26.4 (21.8; 33.2)
Prepregnancy BMI > 30 kg/m^2^ (%)	37.4%
Chronic arterial hypertension (%)*	9.7%
Gestational hypertension/preeclampsia (%)*	16.2%
Women with hyperglycaemia detected in pregnancy (%)	59.7%
Gestational age at the diagnosis of HiP (gestational weeks)	24.0 (13.0; 28.0)
Maternal body weight at the diagnosis of HiP (kg)**	92.0 (80.1; 107.0)
75 g OGTT fasting [mg/dL]^**^	104.5 (95; 118.5)
75 g OGTT 60′ (mg/dL)**	192.0 (182.5; 222.5)
75 g OGTT 120′ (mg/dL)**	159.5 (142.2; 186.7)
Women with severe hyperglycaemia (DiP) (%)*	11.7%
Women treated with diet + insulin (%)*	58.2%

^*^Out of patients with hyperglycaemia diagnosed in pregnancy **Data given for patients with hyperglycaemia diagnosed in pregnancy only

*BMI* body mass index, *HiP* hyperglycaemia detected in pregnancy, *OGTT* oral glucose tolerance test, *DiP* diabetes in pregnancy

Table 2 presents the frequency analysis of the studied polymorphisms in the cohort. Comparing normoglycemic versus the HiP arm of the cohort, we confirmed a statistically significant difference in the distribution of the genotypes of the *GR* rs41423247 polymorphism and a trend for a different distribution of the genotypes of an rs6195 polymorphism. No differences were found for the rs1690 and rs6189 variants of the *GR* gene.

**Table 2:** Genotype and allelic frequency analysis

	Controls		Hyperglycaemia in pregnancy		*p*
	Observed value *n* (%)	Expected value (%)	Observed value *n* (%)	Expected value (%)	
Genotype	*GR* 646C > G (rs41423247)				*** < 0.001***
CC	45 (33.8)	30.6	26 (13.1)	14.5	
CG	57 (42.9)	49.4	99 (50.1)	47.2	
GG	31 (23.3)	20.0	73 (36.8)	38.3	
Total	133 (100%)	100.00	198 (100)	100.00	
Alleles					
C	147 (55.3)	–	151 (38.1)	–	
G	119 (44.7)	–	245 (61.9)	–	
Total	266 (100.00)	–	396 (100.00)	–	
Genotype	*GR* N363S (rs6195)				0.068
AA	125 (94.0)	94.1	176 (88.9)	89.1	
AG	8 (6.0)	5.8	22 (11.1)	10.6	
GG	0 (0)	0.1	0 (0)	0.3	
Total	133 (100%)	100.00	198 (100%)	100.00	
Alleles					
A	258 (97.0)	–	374 (94.4)	–	
G	8 (3.0)	–	22 (5.6)	–	
Total	266 (100.00)	–	396 (100.00)	–	
Genotype	*GR* rs6190				0.524
GG	129 (97.0)	97.0	193 (97.5)	97.4	
GA	4 (3.0)	3.0	5 (2.5)	2.6	
AA	0 (0)	0	0 (0)	0	
Total	133 (100%)	100.00	198 (100%)	100.00	
Alleles					
G	262 (98.5)	–	391 (98.7)	–	
A	4 (1.5)	–	5 (1.3)	–	
Total	266 (100.00)	–	396 (100.00)	–	
Genotype	*GR* rs6189				0.566
GG	129 (97.0)	97.0	189 (95.5)	95.5	
GA	4 (3.0)	3.0	9 (4.5)	4.5	
AA	0 (0)	0	0 (0)	0	
Total	133 (100%)	100.00	198 (100%)	100.00	
Alleles					
G	262 (98.5)	–	387 (97.7)	–	
A	4 (1.5)	–	9 (2.3)	–	
Total	266 (100.00)	–	396 (100.00)	–	

Bolditalic value indicates *p* value < 0.001

Data regarding bivariate associations between the receptor variants and the risk for HiP are summarised in Table 3. We confirmed that the *GG* genotype of the rs41423247 polymorphism is associated with a significantly increased risk for HiP [OR 3.36 (1.95; 5.81), *p* < 0.0001], whereas this risk is significantly reduced in the carriers of the CC genotype of this polymorphism [OR 0.52 (0.31; 0.85), *p* = 0.009]. Both maternal prepregnancy BMI and maternal age were significantly related to the increased risk of HiP: OR 1.27 (1.20; 1.35), *p* < 0.0001, OR 1.13 (1.06; 1.20), *p* = 0.0002, respectively.

**Table 3:** Bivariate analysis of the polymorphisms as predictors for hyperglycaemia detected in pregnancy (HiP); data from bivariate logistic regression

*GR* polymorphism	OR (95%CI) for HiP in the cohort	*p*
rs*41423247CC*	0.30 (0.17; 0.51)	** < *0.001***
rs*41423247CG*	1.32 (0.85; 2.06)	0.219
rs*41423247GG*	1.94 (1.18; 3.18)	***0.009***
rs*6195AG*	1.96 (0.85; 4.55)	0.116
rs*6195AA*	0.51 (0.22; 1.18)	0.116
rs*6195GG*	–	
rs*6190GA*	0.84 (0.22; 3.19)	0.798
rs*6190GG*	1.19 (0.31; 4.52)	0.798
rs*6190AA*	–	
rs*6189GA*	1.54 (0.47; 5.12)	0.478
rs*6189GG*	0.65 (0.19; 2.15)	0.478
rs*6189AA*	–	

Bolditalic value indicates *p* value < 0.001

*GR* glucocorticoid receptor, *HiP* hyperglycaemia detected in pregnancy, *OR* odds ratio, *CI* confidence interval

The association between the *GG* genotype and an increased risk for HiP remained significant in a multivariate analysis (Table 4), including maternal prepregnancy BMI, maternal age, being a carrier of the *GG* and being a carrier of the *CC* variant of the 646C > G polymorphism. This model explained ca. 30% of the variation in the outcome.

**Table 4:** Multivariate logistic regression model for the risk of hyperglycaemia detected in pregnancy (HiP)

Outcome	Predictor	OR (95% CI)	*p*	Nagelkerke *R*^2^
Hyperglycaemia detected in pregnancy	*GR rs41423247GG*	3.09 (1.25;7.64)	0.014	0.299
	Maternal age	1.1 (1.01; 1.19)	0.024	
	Maternal prepregnancy BMI	1.17 (1.09; 1.25)	< 0.0001	

*HiP* hyperglycaemia detected in pregnancy, *GR* glucocorticoid receptor, *BMI* body mass index, *OR* odds ratio, *CI* confidence interval

In the additional analysis of only HiP patients, we confirmed that carriers of the *CC* genotype did not differ in regards to gestational age at the diagnosis of the condition [OR for being diagnosed before the 24th week of gestation: 0.65 (0.23; 1.85), *p* = 0.410], or severity of the disease [OR for being diagnosed with DiP: 1.45 (0.45; 4.67) *p* = 0.529, OR for insulin treatment: 1.73 (0.71; 4.21) *p* = 0.223].

To investigate whether adding a genotype variant to the model improves prediction of HiP as compared to the standard maternal characteristics predictive of the condition, we compared ROC characteristics for Model 1 which included maternal age and prepregnancy BMI. Alternatively, Model 2 was obtained from the multivariate logistic regression, which included maternal age, prepregnancy BMI, *CC* variant of rs41423247 and *GG* variant of rs41423247 polymorphism (Table 4). Results of the analysis are presented in Table 5. Neither of the two models differed significantly regarding their AUC, sensitivity and specificity. Furthermore, each model’s effect size calculated using standardized ORs was small: 0.287 for Model 1 and 0.209 for Model 2.

**Table 5:** Parameters of the regression models with (Model 2) vs. without (Model 1) allelic variants of the rs41423247 polymorphism

Model	AUC (95% CI)	Sensitivity (%)	Specificity (%)	*p* for the model	*p* for the comparison between the models
Model 1	0.79 (0.73; 0.84)	75.4	77.3	** < *0.0001***	0.484
Model 2	0.80 (0.74; 0.85)	86.9	63.6	** < *0.0001***	

Bolditalic value indicates *p* value < 0.001

Moreover, Model 1 was a stronger predictor for HiP than Model 2: OR 3.32 (1.9, 5.8) versus OR 2.39 (1.6; 3.5), respectively.

## Discussion

To the best of our knowledge, our study is the first cohort study reporting an association between a genetic variant in the glucocorticoid receptor associated with increased sensitivity to endogenous glucocorticoids and an elevated risk for hyperglycaemia diagnosed in pregnancy.

Clinical observations regarding an association between allelic variants of a BclI region of the *GR* gene and the metabolic traits related to an increased risk of T2DM consistently report an association between this polymorphism as well as various indirect indicators of insulin resistance: increased abdominal fat, increased waist-to-hip ratio or increased leptin [22]. Interestingly, these associations seem to be unmediated by obesity as results regarding BMI and polymorphism of the BclI region remain inconclusive [22].

Pregnancy itself is a condition characterized by natural insulin resistance. Impaired response to the hormone is necessary to secure foetal priority regarding access to the nutrients (mostly glucose) taken through food; it also promotes maternal energy storage to prepare for increased energy expenditure during breastfeeding. Serious insulin resistance—beyond the compensatory capability of the pancreatic beta-cells—is indicated as a main pathomechanism of HiP. Thus, the carriers of these allelic variants might be predisposed to hyperglycaemia diagnosed in pregnancy due to increased insulin resistance. Contrary to the findings discussed above, a study of 40 case–control pairs with or without metabolic syndrome confirmed that all cases of the *GG* genotype of the rs41423247 polymorphism occurred in healthy controls [23]. This observation might suggest a protective role of this variant against insulin resistance, as metabolic syndrome constitutes a clinical manifestation of this condition. However, the cohort was small and consisted of both male and female patients.

Importantly, Kumsta et al. [24] reported a sexual dimorphism in an activity of the HPA axis in healthy carriers of an rs41423247 *GG* genotype: male carriers showed a reduced ACTH cortisol response to psychosocial stress, whereas female carriers presented a pronounced cortisol reaction to stress tests. These findings support our observation of an elevated HiP risk in the pregnant carriers of this variant. Pregnancy is a challenging period in a woman’s life, and there is strong evidence linking prenatal stress to adverse perinatal outcomes [25]. Data from animal models and clinical observations in humans indicate dysregulation of maternal and foetal HPA axis as a proposed intergenerational transmission mechanism of the stressors’ impact [26, 27]. Some authors also report a negative impact of pregnancy-related stress on women’s glycaemic status [28]. Additionally, a paper from Velders et al. studying mother–offspring pairs from the Generation R study confirmed that genetic variation in the *NR3C1* gene is a driving factor behind the variability of the offspring’s behaviour, and it is linked to maternal psychological symptoms assessed in the mid-pregnancy [29]. Given these results, our study provides valuable data regarding the possible role of stress as a trigger factor mediating genetic susceptibility and certain metabolic phenotypes. The above-mentioned findings reported by various groups also support our observation that HiP related to the *GR* variants may develop due to a combined impact of genetic susceptibility, stress or other environmental factors. However, our initial observations would need to be confirmed by appropriately designed prospective trials.

Also, sex-specific differences were noted for an association between the accumulation of subcutaneous fat and the heterozygous carrier status. This relationship was more pronounced in female adolescents than their male counterparts over a 12-year observation period [30]. Moreover, a relationship between hyperinsulinemia and this polymorphism was confirmed in a small cohort of 56 premenopausal obese women, representing a group phenotypically similar to our cohort [31].

Sex-specific differences in response to a glucose load in a hyperglycaemic clamp were also reported for N363S (rs6195) and ER23/22EK (rs6190, rs6189) polymorphisms. In a cross-sectional, multicentre cohort study, van Raalte et al. described reduced disposition index and reduced first-phase glucose-stimulated insulin secretion in female carriers with these variants [32]. This important information suggests that a sex-specific response can develop a similar metabolic phenotype in female carriers of a variant related to either a relative glucocorticosteroid hypersensitivity (N363S) or insensitivity (ER23/22EK). Although this “beta-cell sparing” phenomenon could be considered protective against combined metabolic-mitogenic consequences of hyperinsulinemia, a reduced response to glucose in pregnancy could result in inadequate compensation for a peripheral insulin resistance driven by pregnancy-related hormones, which is necessary to maintain the normoglycemia in a pregnant individual. Thus, we hypothesize that such individuals could develop a hyperglycaemic phenotype in pregnancy mediated by the altered glucocorticosteroid sensitivity. While this mechanism seems plausible, our study did not confirm a significant association between HiP and the N363S and ER23/22EK polymorphisms.

Our study has some limitations. This is a cohort study without a follow-up, taken from the population referred to the academic unit. Therefore, we were not able to track our patients into the postpartum period to confirm whether the allelic configuration of the *NR3C1* gene in had any impact on the results of the postpartum 75 g oral glucose tolerance test. Moreover, as our participants were recruited among pregnant women referred to a tertiary level of care unit, we also need to consider a referral bias towards more severe phenotypes, including women with concomitant disorders such as obesity or hypertension, or severe hyperglycaemia refractory to nutritional therapy. Also, due to the retrospective nature of our research, we were not able to provide any data on insulin resistance or the biomarkers of the HPA axis in the HiP arm and the controls. Our protocol did not involve any metabolic measurements in neonates.

The major strength of our study comes from observing women with HiP as another phenotype related to the genetic variations of the *GR* gene. Our results also add to the body of evidence regarding combined environmental and genetic factors contributing to the metabolic phenotype of the serious and common disorder of pregnancy. Another novel aspect of our study comes from providing data which justify interdisciplinary interventions; these would not only focus on the glycaemic control but also target HPA axis regarding response to stress. Our observations indicate that the *GR* rs41423247*GG* variant triples the odds of developing HiP for each maternal age and BMI. Therefore, our results could provide a basis for the testing of novel long-term strategies to reduce the risk of hyperglycaemia in pregnancy among the non-pregnant population with lifestyle modifications targeting activity of adrenal axis.

Our study also provides data regarding a serious challenge for public health. An increased amount of evidence consistently reports a rising burden of the long-term metabolic consequences of HiP that is noticeable for both the generations of women of procreative age and their children [33]. A strong relationship between a history of HiP and premature onset of type 2 diabetes mellitus has been reported since the middle of the twentieth century, and this association prompted common testing for the condition in the pregnant population [34]*.* New epidemiological data reported premature cardiovascular morbidity increasing independently from abnormal glucose in women with a history of HiP [35, 36]. Recently, a growing body of evidence describes HiP as a significant contributor to the intergenerational transmission of non-communicable disorders [37]. According to the observational studies, gestational hyperglycaemia in the mother is associated with unfavourable long-term health prognosis for the offspring, particularly regarding early onset obesity or prediabetes/type 2 diabetes [38].

To conclude, our study contributes to the body of evidence on the complex background of a metabolic condition which, although diagnosed in pregnancy, confers the risk of the premature onset of non-communicable disorders in young women and their offspring. Our data also open areas for further research on genetic variants of the *GR* and their impact on a metabolic profile in pregnancy, including maternal response to various pregnancy-related stressors. Such studies should also include a long-term follow-up of mother–child dyads. Future studies should seek to investigate pregnancy-related stress and personality traits as well as their association with metabolic outcomes and complications in pregnancy across the allelic variants of the rs41423247 polymorphism. This should help elucidate an impact of genetic risks versus environmental risks on the development of HiP.

## Data Availability

The data presented in this study are available on request from the corresponding author.
